# Low back pain among nurses working in clinical settings of Africa: systematic review and meta-analysis of 19 years of studies

**DOI:** 10.1186/s12891-020-03341-y

**Published:** 2020-05-16

**Authors:** Ayele Semachew Kasa, Yinager Workineh, Emiru Ayalew, Worku Animaw Temesgen

**Affiliations:** 1 Department of Adult Health Nursing, College of Medicine and Health Sciences, Bahir Dar University, Bahir Dar, Ethiopia; 2 Department of Child Health Nursing, College of Medicine and Health Sciences, Bahir Dar University, Bahir Dar, Ethiopia

**Keywords:** Low back pain, Nurses, Africa, Musculoskeletal problems, Back hygiene

## Abstract

**Background:**

Nurses in Africa are arguably the most important frontline healthcare workers available in most healthcare facilities, performing a broad range of tasks. Such tasks are considerably presumed in the causation of workload. Nursing is listed among the highly risky professions for developing low back pain. The nursing profession is ranked within the top ten professions which have a great risk of low back pain. Hence, this review aimed to ascertain whether low back pain is a significant concern for nurses in African healthcare facilities.

**Methods:**

A comprehensive literature search of different databases with no date limit was conducted from September to November 2018 using the PRISMA guideline. The quality of the included studies was assessed using a 12-item rating system. Subgroup and sensitivity analyses were performed. Cochran’s Q and the I^2^ test were used to assess heterogeneity. The presence of publication bias was evaluated by using Egger’s test and visual inspection of the symmetry in funnel plots.

**Result:**

In this review, 19 studies from different African regions with a total sample size of 6110 nurses were included. All the studies were carried out between 2000 and 2018. Among these, the lowest and the highest prevalence were found to be 44.1 and 82.7% respectively. The estimation of the prevalence rate of low back pain among nurses using the random-effects model was found to be 64.07% (95% CI: 58.68–69.46; *P*-value < 0.0001). Heterogeneity of the reviewed studies was I^2^ = 94.2% and heterogeneity Chi-squared = 310.06 (d.f = 18), *P*-value < 0.0001. The subgroup analyses showed that the highest prevalence of LBP among nurses was from West African region with prevalence rates of 68.46% (95% CI: 54.94–81.97; *P*-value < 0.0001) and followed by North Africa region with prevalence rate of 67.95% (95% CI: 55.96–79.94; P-value < 0.0001).

**Conclusion:**

Even though the overall prevalence of the present study is lower when compared to the Western and Asian studies, it indicated that the prevalence of low back pain among nurses is substantial.

## Background

Low back pain (LBP) is one of the most common causes of musculoskeletal disorders [1]. It is a neglected health problem responsible for serious suffering and disability among nurse professionals [2]. LBP accounted for an average number of disability-adjusted life years (DALYs) higher than different infectious diseases, non-communicable diseases, and road traffic injuries. According to the Global Burden of Disease (GBD) 2010, LBP was reported among the top ten high burden diseases and injuries [3].

Due to the nature of their work healthcare providers are prone to experience lower back pain. On this regard, hospital nurses are groups of healthcare workers who suffered a lot from it [4–6]. The incidence varies between professions and countries [7, 8]. The incidence of LBP being considerably high among nurses working in many healthcare facilities mainly in hospital settings [9].

Nurses are arguably the most important frontline healthcare professionals available in most African healthcare facilities, performing a broad range of tasks. They carried out their activities in settings where no other health workers are available [10]. Such tasks are considerably presumed in causing workload. Due to these and other reasons, nursing is listed among the highly risky professions to experience LBP. In line with this, the nursing profession is ranked within the top ten professions which have a great risk of LBP [11–14].

In their day to day practice, nurses are subjected to lift and transport patients or equipment. They often perform such tasks in difficult environment particularly in developing nations where lifting aids are not available or practicable [15–19]. Such tasks bring a strenuous effect on the back and leads to nurses to experience different musculoskeletal complaints [20]. Biomechanical investigations reported that much strenuous activities on the back results in high spinal load [21].

Low back pain affects nurses’ productivity at work and consequently reduces the overall quality of healthcare the clients receive [22–27]. In addition, LBP will have many negative impact on different aspects of the healthcare system including healthcare workers’ absence from workplace, loss of optimal performance, low job satisfaction, rising medical costs and occupational disability [28].

A survey study on nurses revealed that hospital staff nurses lost 750,000 days a year as a result of back pain [18]. A study done in America with regards to workdays lost due to LBP revealed nurses were ranked the sixth highest to lose their working days from a job [29].

To the researchers’ knowledge, no prior systematic review and meta-analysis work on the prevalence of LBP among nurses in Africa. Hence, the objective of the current review was to thoroughly evaluate peer-reviewed published studies on the reported prevalence of LBP among nurses working at different African healthcare facilities. This would help us to ascertain whether LBP is a significant concern for nurses in the African healthcare facilities.

## Method

This systematic review and meta-analysis was conducted using studies that addressed low back pain among nurses working at different African healthcare facilities. The review was presented using the PRISMA guideline [30].

### Search strategy

To conduct this study, all potentially relevant articles and grey literatures were comprehensively searched from September to November 2018 with no date limit. PubMed, Web of Science, SCOPUS, Science Direct, Google Scholar, CINHAL, ProQuest, African Index Medicus (AIM) and African Journals Online databases were searched using the following search terms: “Burden”, “Magnitude”, “Prevalence”, “Incidence” “Low back pain”, “Musculoskeletal problem”, “Back hygiene”, “Nursing”, “Nurses”, “Professional nurses”, “Registered nurses”, “Hospitals”, “Healthcare facilities” and “Africa”. Search strings were developed using “AND” and “OR” Boolean operators. In addition to the electronic database searches, a secondary search using the list of cited references from the included studies was also considered to identify additional articles.

### Eligibility criteria

Primary researches reported the prevalence of LBP among nurses working at different African healthcare facilities using a 12-month recall period were included. Studies were not restricted by time of study but they should be written or published using English language. Thesis reports, dissertations, and proceedings/conferences that reported the outcome variable were also considered in our search. Studies were excluded if the article was program evaluation, not full text and not published using English language.

### Operational definition

Musculoskeletal disorders: They are described as any pain and/or discomfort that affect the human body’s movement or musculoskeletal system. Low Back Pain (LBP) was operationally defined a pain in the lower back between L1 - L5 and L5-S1 [31]. Prevalence of low back pain: A 12-month recall period was used for experiencing low back pain, as this has been shown to be an appropriate time-scale in other studies [32].

### Data extraction

The title and abstract of the studies were screened based on the preset criteria. Retrieved articles were assessed based on their title, overall objectives, and methodology. Irrelevant and duplicate articles were removed and the full text of the remaining articles that fulfilled the preset criteria were reviewed for inclusion.

To extract the data, a form was prepared that contains: Author names, year of publication, country, region in the continent, setting, study design, sample size, gender, mean age, measurement, and prevalence of LBP. The extraction was done by three independent researchers (ASK, YW, and EA). When there was disagreement between them, a thorough discussion was made and if there was still any disagreement, the fourth author (WA) was consulted.

### Study quality assessment

To assess the quality of the included studies, in the current study a modified critical appraisal tool was utilized. This tool comprises three methodological tests encompassing 12 distinct conditions for prevalence studies. Three questions assess sample representativeness of the target population, six questions assess data quality, and the remaining three questions assess the definition of the outcome variable. Based on this, studies having at least 75% of the total score were acceptable [31, 33–35] to be included in the systematic review and meta-analysis (Appendix).

### Statistical analysis procedure

Data analysis were performed using STATA version 11 software and *P*-value < 0.05 significance level was considered. The weight given to each study was assigned according to the inverse of the variance. Cochrane Q and I^2^ statistics were used to assess heterogeneity among studies. Heterogeneity was measured by I^2^ and divided into four categories; no heterogeneity (0%), low (25–50%), moderate (50–75%), and high (> 75%) [36].

Subgroup analysis and meta-regression (the relationship between the years of the study and region in the continent with the prevalence rate) were employed to explore the cause of heterogeneity between studies. Funnel plot (Begg’s test) and Egger’s statistics with pseudo 95% confidence interval were used to examine publication bias.

## Result

**Fig. 1:**
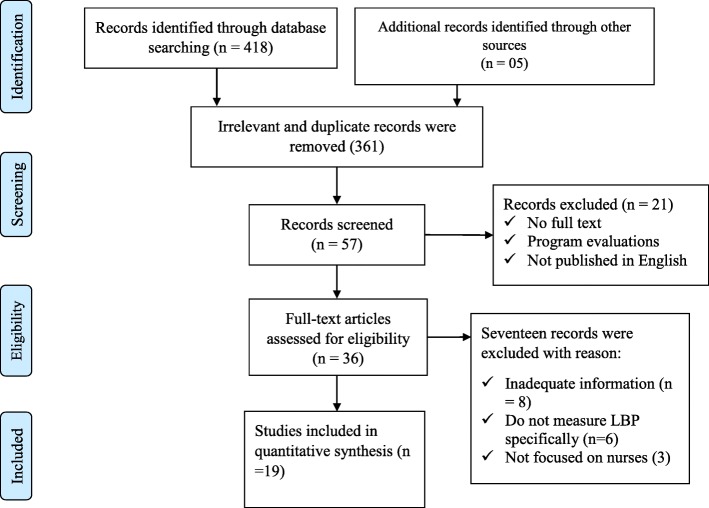
PRISMA flow diagram on prevalence of Low Back Pain (LBP) among nurses working at different healthcare facilities in Africa, 2018

Until December 10, 2018 418 articles were identified. All articles were reviewed and 361 irrelevant and duplicate studies were excluded. The full texts of the remaining 57 articles were reviewed in detail. Finally, 19 articles that met the inclusion criteria were included in the final analysis (Fig. 1).

### Critical appraisal result of the included studies

Criterion number 8 and 9 in the selected critical appraisal instrument were not applicable for most studies except studies done by [37] as they utilized both interview and physical examination techniques to gather the data. Studies were done by [25, 38, 39] utilized both self-administered questionnaire and physical examination so that they utilized criterion number 9 as a critical appraisal. All the included studies for this systematic review and meta-analysis were methodologically assessed and they satisfied the indicated criteria (Table 1).

**Table 1:** Critical appraisal result of the included studies, 2018

Included articles	Criterion No.												
	1	2	3	4	5	6	7	8	9	10	11	12	%
Thembelihle D. et al. [40]	X	✓	✓	✓	✓	✓	✓	NA	NA	X	✓	✓	80
Asmare Y. et al. [41]	✓	X	✓	✓	✓	✓	✓	✓	✓	X	✓	✓	83
M M. Belay et al. [42]	✓	✓	✓	✓	✓	✓	✓	NA	NA	X	✓	✓	90
Lamina S. et al. [21]	X	X	✓	✓	✓	✓	✓	NA	NA	X	✓	✓	70
Lamina S. et al. [21]	X	X	✓	✓	✓	✓	✓	NA	NA	X	✓	✓	70
F. O. Omokhodion et al. [26]	X	✓	✓	✓	✓	✓	✓	NA	NA	X	✓	✓	80
Sikiru L & Hanifa S [43]	X	✓	✓	✓	✓	✓	✓	NA	NA	✓	✓	✓	90
Muhammed A. et al. [28]	✓	X	✓	✓	✓	✓	✓	NA	NA	X	✓	✓	80
Mukaruzima Lela [44]	✓	✓	✓	✓	✓	✓	✓	NA	NA	X	✓	✓	90
Thembelihle D [45].	✓	X	✓	✓	✓	✓	✓	NA	NA	X	✓	✓	80
Chandeu Mwilila [46]	✓	✓	✓	✓	✓	✓	✓	NA	NA	✓	✓	✓	100
Wided B. et al. [31]	✓	X	✓	✓	✓	✓	✓	NA	✓	X	✓	✓	81.8
Ian G Munabi. et al. [47]	✓	X	✓	✓	✓	✓	✓	NA	NA	X	✓	✓	80
Mengestie M. et al. [42]	✓	X	✓	✓	✓	✓	✓	NA	NA	X	✓	✓	80
Betty C [26].	✓	X	✓	✓	✓	✓	✓	NA	NA	X	✓	✓	80
Amany M. et al. [48]	X	X	✓	✓	✓	✓	✓	NA	✓	✓	✓	✓	81.8
Ziadi B. et al. [49]	✓	X	✓	✓	✓	✓	✓	NA	NA	X	✓	✓	80
Bolanle MS. et al. [50]	✓	✓	✓	✓	✓	✓	✓	NA	✓	X	✓	✓	91
Chiwaridzo et al. [51]	✓	X	✓	✓	✓	✓	✓	NA	NA	X	✓	✓	80

*√* criterion fulfilled, *X* criterion not fulfilled *NA* not applicable

Based on the inclusion criteria, 19 studies [15, 20, 22, 25, 37–39, 52–62] were included in the final analysis. All the studies were done using a cross-sectional study design. Even if some studies [15, 22, 25, 61] failed to report the number of male and female participants clearly most of the study participants were females. One study indicated that only female nurse participants were included to their study [38]. The sample size of the studies ranged between 80 from a study done in Nigeria [54] and 880 from Uganda [59]. Concerning to the study facility, almost all studies were done among hospital nurses. Whereas one study from Ethiopia included nurse participants from both hospitals and health centers [37] (Table 2).

**Table 2:** Characteristics of included articles in the systematic review and meta-analysis, 2018

Authors name	Year of publication	Country	Region in the continent	Setting	Study design	Sample size	Sampling method	Measur-ement	Gender (%)	Mean age	Response rate (%)	No. of people with the outcome	Preval-ence (%)
Thembelihle D. et al	2018	South Africa	South	Regional Hospital	cross-sectional	373	Convenience	SSAQ	89% Female	NS	65.0	157	**59**
Asmare Y. et al.	2015	Ethiopia	East	Public Hospital & HC	cross-sectional	428	Survey	A-NMSQ	53.7% Female	30	91%	222	57.1
M M. Belay et al.	2016	Ethiopia	East	Public Hospital	cross-sectional	430	SRS	SSAQ + VAS	72.2% Female	30.6	92%	181	45.8
Lamina S. et al.	2009	Ethiopia	East	Public Hospital	cross-sectional	120	Convenience	SSAQ	NS	NS	83%	60	60
Lamina S. et al.	2009	Nigeria	West	Specialized hospital	cross-sectional	500	Convenience	SSAQ	NS	NS	82%	300	73.5
F. O. Omokhodion et al.	2000	Nigeria	West	Rural hospital	cross-sectional	80	Convenience	SSAQ	33.8% Female	43.8	93%	51	69
Sikiru L & Hanifa S	2010	Nigeria	West	Specialized hospital	cross-sectional	500	VB	SSAQ	63.7% Female	39.2	82%	300	73.5
Muhammed A. et al	2015	Nigeria	West	UTH	cross-sectional	120	multi- stage	SSAQ	NS	NS	82%	81	82.7
Mukaruzima Lela	2010	Rwanda	East	Military Hospital	cross-sectional	133	SRS	IPAQ+NMDQ	82% Female	34.5	82%	88	78
Thembelihle D.	2010	South Africa	South	Public Hospital	cross sectional	373	SRS	SSAQ	79.3%Female	NS	72%	158	59
Chandeu Mwilila	2008	Tanzania	East	MOI	cross sectional	312	Purposive	SSAQ	83.6%Female	35.9	54%	126	73.6
Wided B. et al	2017	Tunisia	North	Teaching hospital	cross-sectional	329	Survey	Borg CR-10 scale, JCQ	NS	39.8	61.70%	125	58.1
Ian G Munabi. et al.	2014	Uganda	East	Public Hospital	cross-sectional	880	Survey	DMQ & NMDQ	85.7%Female	35.4	85.40%	459	61.9
Mengestie M. et al.	2016	Ethiopia	East	Public Hospital	cross-sectional	395	SRS	SSAQ	72.2%Female	30.6	91.9	166	45.8
Betty C.	2015	Kenya	East	Both public & private hospitals	cross-sectional	169	SRS	SSAQ	76.9%Female	35	76.9	100	76.9
Amany M. et al.	2014	Egypt	North	Public Hospital	cross-sectional	150	Purposive	OLBDQ	100%Female	NS	100	119	79.3
Ziadi B. et al.	2014	Algeria	North	Public Hospital	cross-sectional	450	SRS	SSAQ	NS	NS	66.7	200	66.7
Bolanle MS. et al.	2010	Nigeria	West	UH,GH,PH	cross-sectional	160	SRS	SSAQ	97.5%Female	36.4	80	56	44.1
Chiwaridzo et al.	2018	Zimbabwe	East	Public Hospital	cross-sectional	208	Stratified RS	NMDQ	84.6%Female	32	55.7	65	55.7

*A-NMSQ* adapted from nordic musculoskeletal questionnaires, *DMQ* Dutch musculoskeletal questionnaire, *GH* General Hospital, *HC* Health Center, *IPAQ* International Physical Activity Questionnaire, *JCQ* job content questionnaire, *MOI* Muhimbili Orthopedic Institute, *NMDQ* Nordic musculoskeletal disorder questionnaire, *NS* not stated, *OLBDQ* Oswestry low back disability questionnaire, *PH* Private Hospital, *RS* random sampling, *SRS* systematic random sampling, *SSAQ* standardized self-administered questionnaire, *UH* University Hospital, *UTH* University Teaching Hospital, *VB* volunteer based and *VAS* visual analog scale

### Prevalence of low back pain (LBP)

**Fig. 2:**
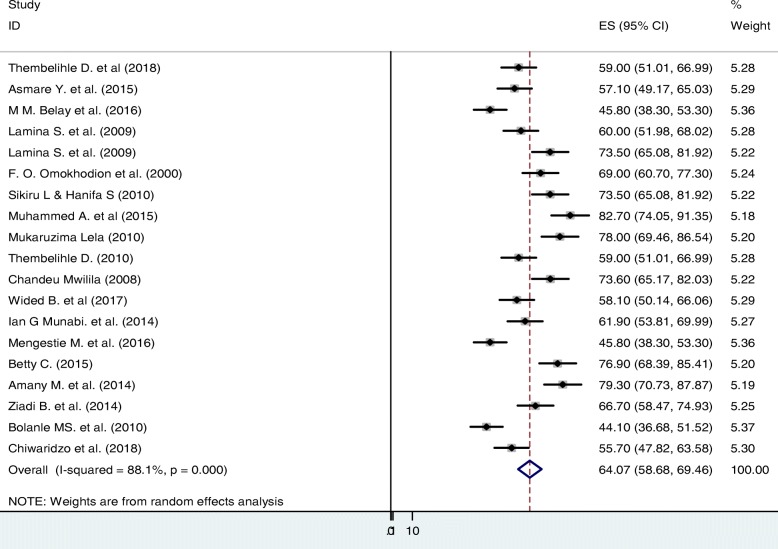
Forest plot of prevalence of low back pain among nurse in the African healthcare facilities, 2018

In this review, 19 studies from different African regions with a total sample size of 6110 nurses were included. All the studies were carried out between 2000 and 2018. From these 19 studies, the lowest and the highest reported prevalence of LBP were 44.1% [39] and 82.7% [22] respectively. Both the highest and the lowest prevalence of LBP were reported from a study done in Nigeria. The estimation of the prevalence rate of LBP among nurses using the random-effects model was 64.07% (95% CI: 58.68–69.46; *P*-value < 0.0001). Heterogeneity of the reviewed studies was I^2^ = 94.2% and heterogeneity Chi-squared = 310.06 (d.f = 18), P-value < 0.0001 (Fig. 2).

### Subgroup analysis

**Fig. 3:**
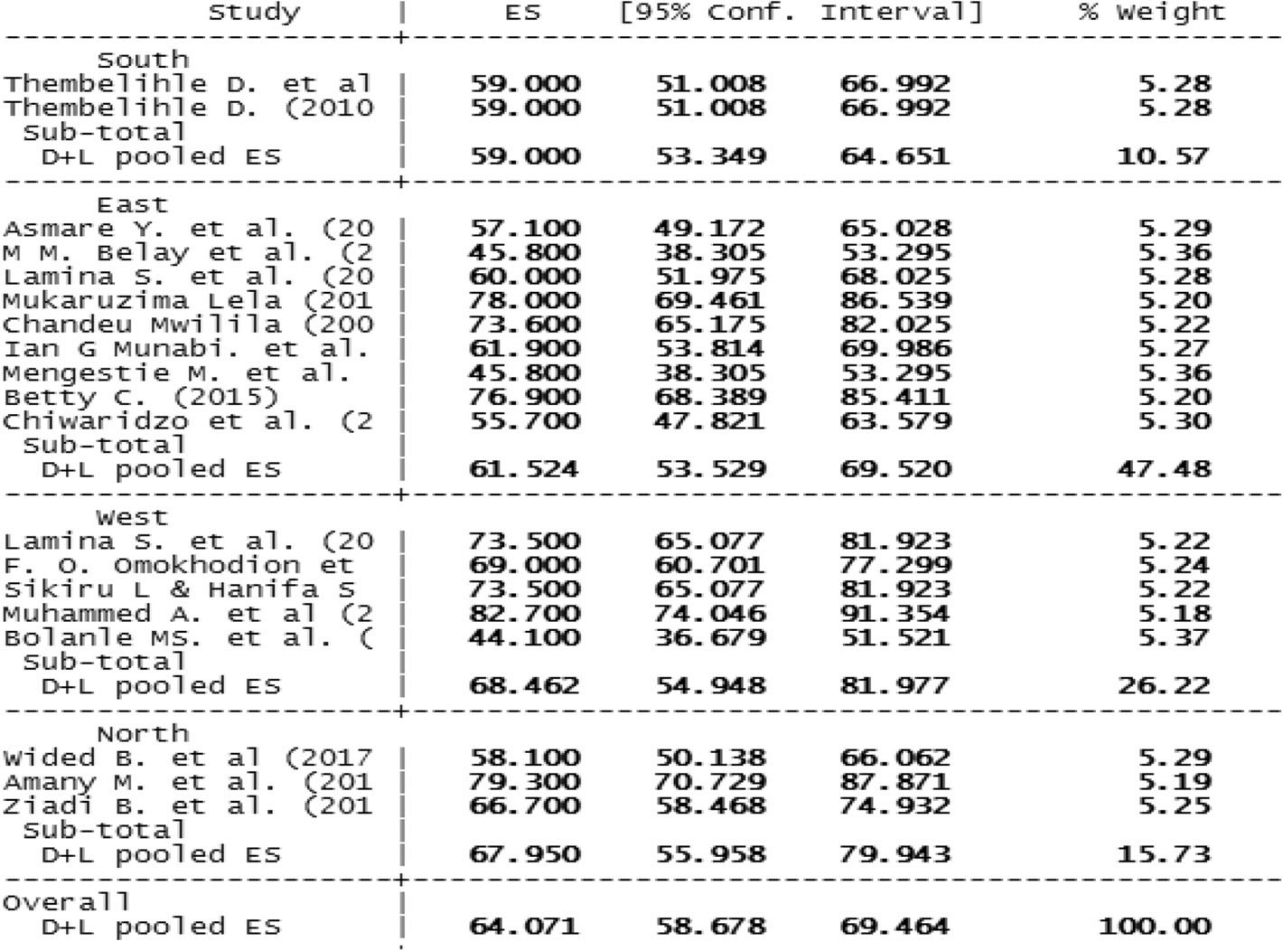
Subgroup analysis of low back pain among nurse using region of the continent in the African healthcare facilities, 2018

According to the subgroup analyses, the highest prevalence of LBP among nurses was reported from West African region with prevalence rates of 68.46% (95% CI: 54.94–81.97; *P*-value < 0.0001) followed by North Africa region with prevalence rate of 67.95% (95% CI: 55.96–79.94; P-value < 0.0001). These two African regions had the highest prevalence of LBP as compared to their South African counterparts, 59.00% (95% CI: 53.34–64.65; *P*-value < 0.0001) (Fig. 3).

### Meta –regression

**Fig. 4:**
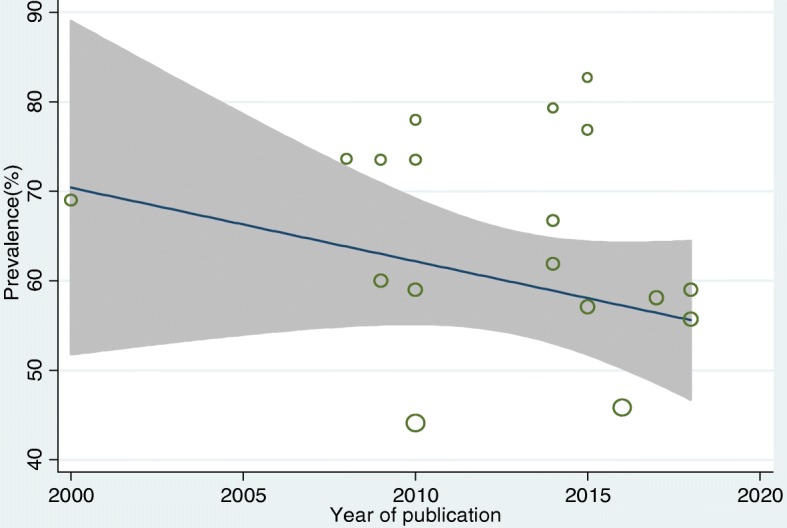
Funnel plot showing the relation between year of publication and prevalence of LBP among nurses working in different African health facilities, 2018

Meta-regression analysis showed that there was no significant statistical relationship between the year of publication and the prevalence of the LBP (β = − 0.82, P-value = 0.808) (Fig. 4).

**Fig. 5:**
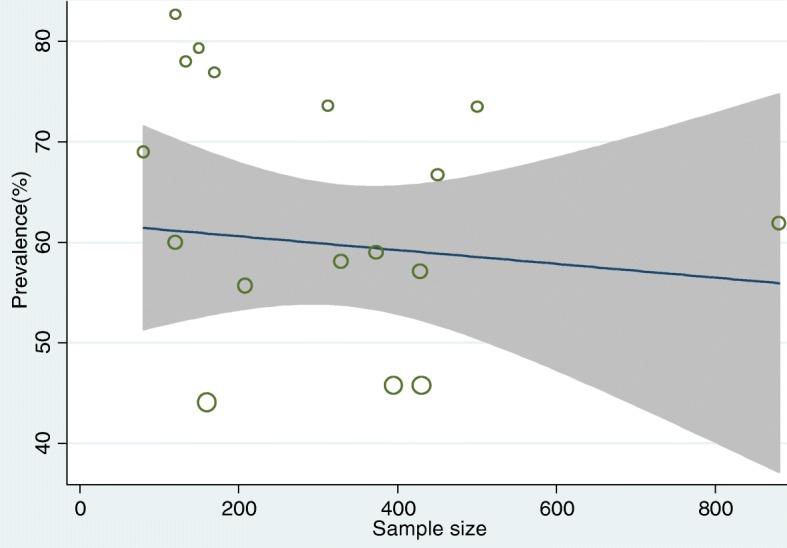
Funnel plot showing the relation between sample size and prevalence of LBP among nurses working in different African health facilities, 2018

The meta-regression also showed that there was no significant statistical relationship between the sample size and the prevalence of LBP (β = − 0.007, P-value = 0.93) (Fig. 5).

**Fig. 6:**
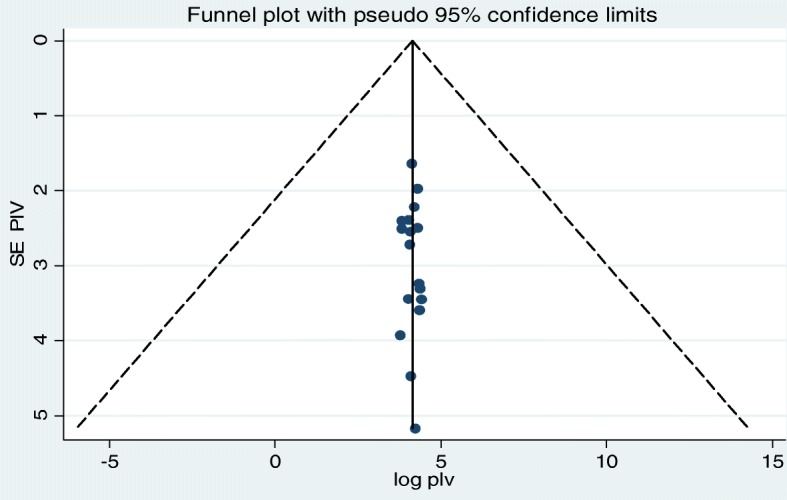
Funnel plot for assessing publication bias among studies

To assess publication bias, the funnel plot and Egger’s test were conducted in the meta-analysis. The funnel plot and Egger’s regression tests (β = − 0.0024, SE = 0.06, *P* = 0.96) showed that no evidence of publication bias for the included studies (Fig. 6).

## Discussion

Low back pain is a common work-related musculoskeletal disorder in healthcare workers. Particularly, it imposes high risk on nursing professionals working at different healthcare facilities mainly in hospital settings [63, 64]. Different studies have shown nursing personnel had higher prevalence of LBP relative to the general population or other occupational groups [41, 48, 65]. Such problems are reported in influencing the quality of life of healthcare professionals. This will, in turn, affects the healthcare quality [23].

This study denotes the first effort to report the prevalence of LBP among nurses working in healthcare facilities in the African continent. Hence, the aim of this systematic review and meta-analysis was to determine the pooled prevalence of LBP among nurses working at different healthcare facilities in African regions. By providing a comprehensive picture, this study would help to recognize the impact of the problem on nurse professionals in African countries.

Low back pain is a common occupational problem for nurses worldwide. LBP has been previously reported at rates between 45% in England [50], 63% in Australia [40]. Researches from Hong Kong and China also showed that nurses experience LBP about 40.6% [42] and 56% [26] respectively. Different African studies reported that LBP rates as 44.1, 79.4 and 82.7% [22, 39, 43, 44].

Literatures stated that the 12-month prevalence of LBP among nursing personnel is estimated to be up to 65% [45]. The result of the present systematic review and meta-analysis also showed that the overall 12-month prevalence of LBP among nurses was 64.07%. This finding was higher than from studies done in Iran that showed the overall prevalence of LBP among nurses was 61.2 and 60.98% respectively [31, 46]. Whereas studies in the Western nations and Asian countries revealed that the overall prevalence of LBP among nurses was higher compared to the present finding. Studies from Japan [47], Turkey [8] and the United States of America [49] showed that the overall prevalence of LBP among nurses was 91.9, 77.1 and 72.5% respectively. All the studies revealed that the existence of higher prevalence of LBP. Studies done in Switzerland [51] and Italy [66] also revealed that the overall annual prevalence of LBP among nurses was 73–76 and 86% respectively. This also confirms that there is a higher prevalence of LBP among nurses in the Western nations.

Studies done in the Western and in some Asian countries revealed that there is a higher prevalence of LBP among nurses. This finding is confirmed by different literatures in the subject area. A systematic review on LBP among Asian nurses revealed that the overall prevalence was 71.85% [67]. This high existence of LBP among nurses in the developed and in some Asian nations might be due the existence of high workload [68] for patient care, conducting advanced procedures that require prolonged standing. Such tasks all might lead nurses to experience LBP in their working environment. Studies also indicated that the prevalence of LBP was linked with both demographic characteristics of nurses, psychological factors and hospitals’ organizational factors [69]. In addition, the variation of the prevalence of LBP among studies might be accounted by variations in the tool utilized in assessing the outcome variable.

The results of this study identified the presence of a high prevalence of LBP among nurses in the Western region of Africa. In the present study, five different studies were incorporated from the Western region of Africa, all of them were from Nigeria, a country with the highest population in the continent. Coupled with a high population, with many patients and no enough healthcare providers these all will have their own impact on the countries healthcare system [70]. A report by Good Health Weekly revealed that Nigerian health professionals mainly nurses are experiencing high workload, burnout, stress, and demotivation from their work [70]. This all will cause nurses to experience LBP in one or in another way. Moreover, in the studies conducted in Nigeria, nurses were included from specialized and teaching hospitals. This, in turn, will have an additional workload on nurses that will expose them to suffer from LBP. The current review revealed variations in LBP among nurses working in the four regions of Africa. The variation was due to differences on having high client flow to the healthcare system [70] and nurses providing a broad range of tasks in such regions. But further researches are imperative to find out the possible route cause of LBP in each specific regions of Africa.

As mentioned in many literatures, nurses are the number one frontline healthcare professionals who contact clients in a variety of healthcare setups. This would have its own share to experience workload and consequently, they will be at higher risk of developing LBP.

### Strength and limitation

The search was not restricted by time of study or year of publication. In addition, all possible findings from thesis report, dissertation, and any report proceedings/conferences in the subject matter were considered in our searches. As a limitation, adequate studies were not incorporated from some regions of the continent and even most of the studies were concentrated in a single country in each region of the continent. This might have its own shortfalls in producing the overall picture of the problem to the continent as a whole.

## Conclusion

The overall prevalence of the present study is lower compared to the Western and Asian studies. The current finding indicated that low back pain is a significant concern amongst nurses in Africa.

The result may be helpful for hospital administrators and other concerned government agencies to implement measures in reducing the incidence of low back pain on nurses. The possible measures will include considering ergonomics solutions, stress reduction strategies, providing training for the nurses that can significantly reduce the risk of experiencing LBP. All the efforts made would improve nurses’ sense of belongingness, retention, quality of patient care and even organizational culture.

## Data Availability

All data are available in the manuscript.

## Appendix

**Table 3:** The critical appraisal tool utilized to assess the quality of the included studies

Part I	Is the final sample representative of the target population?
	1. At least one of the following must apply in the study: an entire target population, randomly selected sample, or sample stated to represent the target population.
	2. At least one of the following: reasons for non-response described, non-responders described, comparison of responders and non-responders, or comparison of sample and target population
	3. Response rate and, if applicable, drop-out rate reported
Part II	Quality of the data?
	4. Were the data primary data of low back pain or was it taken from a survey not specifically designed for that purpose?
	5. Were the data collected from each adult directly or were they collected from a proxy?
	6. Was the same mode of data collection used for all subjects?
	7. At least one of the following in case of questionnaire: a validated questionnaire or at least tested for reproducibility.
	8. At least one of the following in the case of an interview: Interview validated, tested for reproducibility, or adequately described and standardized.
	9. At least one of the following in the case of an examination: Examination validated, tested for reproducibility, or adequately described and standardized.
Part III	Definition of low back pain (LBP)
	10. Was there a precise anatomic delineation of the lumbar area or reference to an easily obtainable article that contains such specification?
	11. Was there further useful specification of the definition of LBP, or question(s) put to study subjects quoted such as the frequency, duration or intensity, and character of the pain. Or was there reference to an easily obtainable article that contains such specification?
	12. Were recall periods clearly stated: e.g., 1 week, 1 month or lifetime?

## References

[1] Cunningham C, Flynn TBC (2006). Low back pain and occupation among Irish health workers. Occup Med.

[2] Kamper SJ (2015). Multidisciplinary biopsychosocial rehabilitation for chronic low back pain: Cochrane systematic review and meta-analysis. Br Med J.

[3] Vos T (2012). Years lived with disability (YLDs) for 1160 sequelae of 289 diseases and injuries 1990–20100: a systematic analysis for the global burden of disease study. Lancet.

[4] Lipscomb J, Trinkoff A, Brady B, Geiger-Brown J (2004). Health care system changes and reported musculoskeletal disorders among registered nurses. Am J Public Health.

[5] Bejia I, Younes M, Hadj J, Khalfallah T, Ben K (2004). Prevalence and factors associated to low back pain among hospital staff. Rev du Rhum.

[6] Wong TS, Teo NKM (2010). Prevalence and risk factors associated with low back pain among healthcare providers in a district hospital. Malays Orthop J.

[7] Johnson OEEE (2016). Prevalence and risk factors of low back pain among workers in a health facility in south-South Nigeria. BJMMR.

[8] Karahan A, Kav S, Abbasoglu A, Dogan N (2009). Low back pain: prevalence and associated risk factors among hospital staff. J Adv Nurs.

[9] Burdorf ASG (1997). Positive and negative evidence of risk factors for back disorders. Scand J Work Env Heal..

[10] Munjanja O, Kibuka SDD (2005). The nursing workforce in sub-Saharan Africa.InThe global nursing review initiative.

[11] Nelson A (2006). Development and evaluation of a multifaceted ergonomics program to prevent injuries associated with patient handling tasks. Int J Nurs.

[12] Hllhlrnakl H (1991). Low-back pain, its origin and risk indicators. Scand J Work Env Heal.

[13] Dlungwane T, Voce A, Knight S (2018). Prevalence and factors associated with low back pain among nurses at a regional hospital in KwaZulu-Natal, South Africa. Heal SA Gesondheid.

[14] Alexandre NMC, Angerami ELS, Moreira FD (1996). Back pain and nursing. Rev da Esc Enferm da USP.

[15] Sikiru L, Shmaila H (2009). Prevalence and risk factors of low Back pain among nurses in Africa: Nigerian and Ethiopian specialized hospitals survey study. East African J Pablic Heal.

[16] Cesana G, Arduca A, Latocca R, SG (1998). Risk evaluation and health surveillance in hospitals: a critical review and contribution regarding experience obtained at Garardo Dei Tintori Hospital in Monza. Med Med –Lav 1998.

[17] Vieira ER, Kumar S, Coury HJCG, Narayan Y (2006). Low back problems and possible improvements in nursing jobs. J Adv Nurs.

[18] Triolo PK (1988). Occupational health hazard of hospital staff nurses. Part II physical, chemical and biological stressors. AAOHN – J.

[19] Gourmelen J (2007). Frequency of low Back pain among men and women aged 30 to 64 years in France. Results of two National Surveys. Ann Réadaptation Médecine Phys.

[20] Tanui BC (2015). Assessment of Work-Related Musculoskeletal Disorders among Nurses in Mombasa County, Kenya.

[21] Tate RB, Yassi A, Cooper J (1999). Predictorsof time loss after Back injury in nurses. Spine (Phila Pa 1976).

[22] Farooq MA, Awwal LM, Musa HA, Mustapha GA (2015). Work-related risk factors for lower Back pain among nurses in Ahmadu Bello University teaching hospital ( ABUTH ), Zaria-Nigeria. IOSR J Nurs Heal Sci.

[23] Punnett L, Wegman D (2004). Work-related musculoskeletal disorders: the epidemiologic evidence and the debate. J Electromyogr Kinesiol.

[24] De Castro A (2004). Handle with care: the American Nurses Association’s campaign to address work-related musculoskeletal disorders. Online J Issues Nurs.

[25] Boughattas W, Maalel O, El MM, Bougmiza I (2017). Low Back pain among nurses: prevalence , and occupational risk factors. Occup Dis Environ Med.

[26] Smith DR, Wei N, Kang L, Wang RS (2004). Musculoskeletal disorders among professional nurses in mainland China. J Prof Nurs.

[27] Holder N (2009). Cause, prevalence, and response to occupational musculoskeletal injuries reported by physical therapists and physical therapist assistants. Phys Ther.

[28] Asadi P, Monsef Kasmaei V, Zia Ziabari SZB (2016). The prevalence of low back pain among nurses working in Poursina hospital in Rasht, Iran. J Emerg Pr Trauma.

[29] Salvi Shah BD (2012). Prevalence of low Back pain and its associated risk factors among doctors in Surat. Int J Heal Sci Res.

[30] Moher D, Liberati A, Tetzlaff J, Altman DGPG. Preferred reporting items for systematic reviews and meta-analyses: the PRISMA statement. PLoS Med. 2009;6(6). 10.1371/journal.pmed1000097. PMC3090117 21603045

[31] Azizpour Y, Delpisheh A, Montazeri Z, Sayehmiri K (2017). Prevalence of low back pain in Iranian nurses : a systematic review and meta- analysis. BMC Nurs.

[32] Chiou WK, Wong MKLY (1994). Epidemiology of low back pain in Chinese nurses. Int J Nurs Stud.

[33] Walker BF (2000). The prevalence of low back pain: a systematic review of the literature from 1966 to 1998. J Spinal Disord.

[34] Mousavi SJ, et al. Low back pain in Iran: a growing need to adapt and implement evidencebased practice in developing countries. Spine (Phila Pa 1976). 2011;36(10). 10.1097/BRS.0b013e3181fa1da2. 10.1097/BRS.0b013e3181fa1da2 21270691

[35] Louw QA, Morris LDG-SK. The prevalence of low back pain in Africa: a systematic review. BMC Musculoskelet Disord. 2007;8(105) Available from: 10. 1186/1471-2474-8-105. 10.1186/1471-2474-8-105 PMC2198912 17976240

[36] Ades AE, Lu GHJ (2005). The interpretation of random-effects metaanalysis in decision models. Med Decis Mak.

[37] Institutions H, Yitayeh A, Mekonnen S, Fasika S, Gizachew M (2015). Emergency medicine: open access annual prevalence of self-reported work related musculoskeletal disorders and associated factors among nurses working at Gondar town governmental health Institutions, Northwest Ethiopia. Emerg Med (Los Angel).

[38] El-soud AMA, El-najjar AR, El-fattah NA, Hassan AA (2014). Prevalence of low back pain in working nurses in Zagazig University hospitals: an epidemiological study. Egypt Rheumatol Rehabil.

[39] Tinubu BMS, Mbada CE, Oyeyemi AL, Fabunmi AA (2010). Work-related musculoskeletal disorders among nurses in Ibadan , south-West Nigeria: a cross-sectional survey. BMC Musculoskelet Disord.

[40] Lusted MJ, Carrasco CL, Mandryk JAHS (1996). Self reported symptoms in the neck and upper limbs in nurses. Appl Ergon.

[41] Matsudaira K, Palmer KTRI (2011). Prevalence and correlates of regional pain and associated disability in Japanese workers. Occup Env Med.

[42] Yip Y (2001). A study of work stress, patient handling activities and the risk of low back pain among nurses in Hong Kong. Aust J Adv Nurs.

[43] TinubuBM MCE, FA OAL (2010). Work-related musculoskeletal disorders among nurses in Ibadan, south-West Nigeria: a cross-sectional survey. BMC Musculoskelet Disord.

[44] Fabunmi AA, Gbiri CA (2008). Relationship between balance performance in the elderly and some anthropometric variables. Afr J Med Med Sci.

[45] Alzahrani H, Mackey M, Stamatakis E, Zadro JR, Shirley D (2019). The association between physical activity and low back pain : a systematic review and meta- analysis of observational studies. Sci Rep.

[46] Soroush A (2018). Musculoskeletal disorders as common problems among Iranian Nurses : a systematic review and Meta - analysis study. Int J Prev Med.

[47] Smith DR, Kondo N, Tanaka E, Tanaka H, Hirasawa K YZ. Musculoskeletal disorders among hospital nurses in rural Japan. Rural Remote Heal J. 2003;241(3):1–7. 15882100

[48] Leighton DJRT (1995). Epidemiological aspects of back pain: the incidence and prevalence of back pain in nurses compared to the general population. Occup Med.

[49] Josephson M, Largerstrom M, Hagberg MHE (1997). Musculoskeletal symptoms and job strain among nursing personnel: a study over a three year period. Occup Env Med..

[50] Smedley J, Egger P, Cooper CCD (1995). Manual handling activities and risk of low back pain in nurses. Occup Env Med..

[51] Maul I, Läubli T, Klipstein AKH (2003). Course of low back pain among nurses: a longitudinal study across eight years. Occup Env Med.

[52] Dlungwane Thembelihle AV& SK. Prevalence and factors associated with low back pain among nurses at a regional hospital in KwaZulu-Natal, South Africa. Heal SA Gesondheid.. 2014;1–6. Available from: http://www.hsag.org.za. Accessed 25 Sept 2018. 10.4102/hsag.v23i0.1082 PMC6917379 31934378

[53] Belay MM, Worku A, SA, BLW G (2016). Epidemiology of low Back pain among nurses working in public hospitals of Addis Ababa, Ethiopia. East Cent African J Surg.

[54] Omokhodion (2000). Prevalence of low Back pain among staff in a rural Hospital in Nigeria. Occup Med (Chic Ill).

[55] Sikiru L, Hanifa S. Prevalence and risk factors of low back pain among nurses in a typical Nigerian hospital. Afr Health Sci. 2010;10(1):26–30. PMC2895788 20811521

[56] Lela M. The relationship between physical activity and low Back pain among nurses in Kanombe military hospital: University of the Western Cape; 2010.

[57] Thembelihle. Prevalence of Low Back Pain Amongst Nurses at Edendale Hospital A dissertation submitted to the Department of Public Health Medicine Nelson R . Mandela School of Medicine Durban , South Africa In partial fulfilment of the requirements for the Master in Pu. University of KwaZulu-Natal; 2010.

[58] Chandeu M. Work-related low Back pain among clinical nurses in Tanzania: University of the Western Cape; 2008.

[59] Munabi IG, Buwembo W, Kitara DL, Ochieng J, Mwaka ES (2014). Musculoskeletal disorder risk factors among nursing professionals in low resource settings : a cross-sectional study in Uganda. BMC Nurs.

[60] Belay MM, Worku A, SA Gebrie BW. (2016). Epidemiology of low back pain among nurses working in public hospitals of Addis Ababa, Ethiopia. East Cent African J Surg..

[61] Boukerma Z, Behlouli AL, Reggad M (2014). Epidemiology of low back pain among nurses of the hospital of Sétif (Algeria). BMJ Occopational Environ Med.

[62] Chiwaridzo M, Makotore V, Dambi JM, Munambah N, Mhlanga M (2018). Work - related musculoskeletal disorders among registered general nurses : a case of a large central hospital in Harare , Zimbabwe. BMC Res Notes.

[63] Wilkinson WE, Salazar MK, Uhl JE, Koepsell TD, DeRoos RLLR (1992). Occupational injuries: a study of health care workers at a northwestern health science center and teaching hospital. Am Assoc Occup Heal Nurs J.

[64] Stubbs DA, Buckle P, Hudson MP, Butler PERP (1983). Back pain in the nursing profession, part I: epidemiology and pilot methodology. Ergon..

[65] Tosunoz IK (2017). Low Back pain in nurses. Int J Caring Sci.

[66] Corona G, Amedei F, Miselli F, Padalino MP, Tibaldi SFG (2005). Association between relational and organizational factors and occurrence of musculoskeletal disease in health personnel. G Ital Med Lav Ergon.

[67] Ellapen TJ, Narsigan S (2014). Work related musculoskeletal disorders among Nurses : systematic review. Ergonomics..

[68] Shieh SH, Sung FC, Su CH, Tsai YHV (2016). Increased low back pain risk in nurses with high workload for patient care: a questionnaire survey. Taiwan J Obs Gynecol.

[69] Soylar P, Ozer A. Evaluation of the prevalence of musculoskeletal disorders in nurses : a systematic review. Med Sci. 2018;7(3):479–85.

[70] Vanguard. Shortage of medical personnel: Tougher times ahead for Nigerians. 2015. Available from: https://www.vanguardngr.com/2015/01/shortage-medical-personnel-tougher-times-ahead-nigerians-1/. Accessed 5 Oct 2018.

